# Peripheral Prosthetic Vascular Graft Infection: A 5-Year Retrospective Study

**DOI:** 10.3390/medsci13020071

**Published:** 2025-06-01

**Authors:** Giovanni De Caridi, Mafalda Massara, Chiara Barilla, Filippo Benedetto

**Affiliations:** 1 Dipartimento di Scienze Biomediche, Odontoiatriche e delle Immagini Morfologiche e Funzionali, Università di Messina, 98122 Messina, Italy; barillachiara@gmail.com (C.B.); dott.filippobenedetto@gmail.com (F.B.); 2 Divisione di Chirurgia Vascolare ed Endovascolare, GOM di Reggio Calabria, 89124 Reggio Calabria, Italy; drmafaldamassara@gmail.com

**Keywords:** critical lower limb ischemia, bypass, negative-pressure wound therapy-peripheral vascular disease-infection

## Abstract

**Background/Objectives:** Peripheral prosthetic vascular graft infection represents a very serious complication after lower limb revascularization, with amputation and mortality rates up to 70% and 30%, respectively. This study was designed to determine the incidence of prosthetic graft infection, amputation, and mortality rate in our institution, analyzing different types of treatment. **Methods:** A retrospective cohort single institution review of peripheral prosthetic bypass grafts evaluated patient demographics, comorbidities, indications, location of bypass, type of prosthetic material, and case urgency and evaluated the incidence of graft infections, amputations, and mortality. **Results:** Between January 2016 and December 2021, a total of 516 bypasses were recorded (318 male, 198 female, mean age 74.2): 320 bypasses in venous material and 196 prosthetic bypasses using Dacron or PTFE. Among patients with a prosthetic bypass, 16 (8.2%) presented a graft infection at a mean follow-up of 39 months. Thirteen other patients who submitted to prosthetic peripheral bypass in other centers presented to our institution with a graft infection, so a total of 29 infected grafts were treated. Infected grafts were removed in 20 patients (68.9%), while a conservative treatment was helpful in nine cases (31.1%). The germs involved were Gram-negative in 27.6% and Gram-positive in 41.4%. During follow-up, we recorded five deaths (17.2%) and six amputations (20.7%) directly after bypass excision; another two amputations (6.9%) occurred after failure of the new bypass replacing the prosthesis removed. **Conclusions:** Redo-bypass, active infection at the time of bypass, and advanced gangrene were associated with a higher risk for prosthetic graft infection and major extremity amputation. Complete graft removal and replacement by venous material or Omniflow II represents the typical treatment. However, aggressive local treatment including drainage, debridement, vacuum-assisted closure therapy application, and muscle transposition seem to be a better solution in selected patients without the need for graft removal and with rates of limb salvage superior to those obtained with excisional therapy.

## 1. Introduction

Peripheral prosthetic vascular graft infection (PVGI) after surgical revascularization for critical lower limb ischemia (CLI) is a serious complication with a reported incidence that ranges from 0.7 to 7% [1,2] and post-operative amputation and mortality rates up to 70% and 30%, respectively [3]. Predictors of surgical site infections after surgical bypass include obesity, diabetes, poor preoperative functional status, a history of smoking, redo-bypass, and female gender [4,5].

Aside from the adverse effect on the patient, post-operative vascular surgical infections significantly contribute to health care costs, as they often require additional procedures and a long hospital stay [6]. The type of treatment depends on the location of the prosthesis, the extent of the infection, the degree of stenosis and/or occlusion, the patient’s general condition, and his life expectancy.

Antibiotic therapy is the first stage of management of PVGI. Excision of the infected prosthetic graft, with or without reconstruction, has been considered conventional therapy. In the absence of appropriate venous graft material, there are prosthetic grafts with a potentially higher resistance to reinfection (cryopreserved arterial allograf, silver-coated grafts, rifampicin-bonded grafts, and bovine-pericardium xenogenous grafts) [7,8,9,10,11]. Although reinfection rates are higher compared to venous grafts and long-term patency is significantly lower than in venous bypasses, the reconstruction is possible by several materials.

It has long been recognized, however, that not all infected bypasses need to be removed. Recent reports have described the treatment of infected lower extremity arterial grafts with aggressive local tissue debridement, administration of local and systemic antibiotics, coverage by muscle transposition [12,13,14,15], as well as graft preservation with the use of vacuum-assisted closure (VAC) devices, with or without muscle flap coverage [16,17,18,19,20,21].

In the present study, we report our personal experience of PVGI management after lower extremity bypass, examining the impact of PVGI on post-operative amputation and death rates.

## 2. Materials and Methods

We performed a retrospective study of all patients presenting a peripheral prosthetic bypass infection in our Institution of Vascular Surgery Division G. Martino University Hospital of Messina, a referral center for CLI treatment.

Between January 2016 and December 2021, a total of 516 bypasses were recorded: 320 bypasses in venous material and 196 prosthetic bypasses using Dacron or PTFE (41 axillo-femoral, 43 femoro-femoral crossover, 27 ilio-femoral, 44 ilio or femoro-tibial, 41 femoro-popliteal bypasses).

A patient was considered as having definite PVGI if at least two of the three following criteria were present: (1) positive bacterial culture of intraoperative specimens or blood samples; (2) clinical signs of infection [general (fever, chills, septic shock) or in the area of the prosthesis (e.g., inflammatory signs in the area of the vascular graft: local pain, erythema or tumefaction, sinus tract infection communicating with PVGI, intraoperative gross purulence or failure of graft consolidation]; (3) biological signs of infection (C-reactive protein > 5 mg/L, white blood count > 12 G/L).

Each case of definite infection was classified as early-onset infection (occurring within 3 months after surgery) or as late-onset infection (occurring more than 3 months after surgery).

## 3. Results

Among 196 prosthetic bypasses performed in our institution, a post-surgical PVGI occurred in 16 cases (8.2%) between 7 days and 3 years after the implantation. Thirteen other patients who submitted to a prosthetic peripheral bypass in other institutions presented at our unit with a PVGI, so a total of 29 cases of prosthetic peripheral graft infection were treated. The infection occurred in 18 males and 11 females, presenting a mean age of 73.9. Follow-up time ranged from 18 months to 5 years and started from the initial operation. Demographic and clinical characteristics of the patients, germs involved, typology of treatment, and outcomes are reported in Table 1. The patients presented the following risk factors and comorbidities: diabetes mellitus (21, 72.4%), hypertension (23, 79.3%), dislipidemia (17, 58.6%), smoke (20, 68.9%), chronic renal failure on hemodialysis (4, 13.8%), chronic obstructive pulmonary disease (11, 37.9%), obesity (9, 31%), coronary artery disease (19, 65.5%). The revascularization procedures performed before the onset of infection are listed in Table 2. The indications were tissue loss (21, 72.4%) and rest pain (8, 27.6%), with 10 (34.5%) redo-bypasses after failure of previous multiple attempts of revascularization through a venous or composite bypass. The infected graft was politetrafluoroethylene (PTFE) in 25 cases (86.2%) and Dacron in four cases (13.8%) (Table 3). The time from prosthesis implantation to development of PVGI was described as early in 18 cases (62.1%) and late in 11 cases (37.9%). The modes of graft infection presentation were as follows: bleeding (four, 13.8%), acute ischemia of the lower limb (three, 10.3%), of which one with associated distal septic skin lesions (22,23), occluded graft (two, 6.9%), false aneurysm (six, 20.7%); suture dehiscence with persistent discharge (nine, 31.1%), abscess (three, 10.3%), seroma (two, 6.9%). Fever > 38 °C was recorded in 21 cases (72.4%), while elevation of white blood cells count (range 12–25), C-reactive protein (range: 5–20), and procalcitonin (PCT) values (range 2–28) was observed in all cases. Aerobic and anaerobic cultures were obtained with swabs from the grafts during operations in 22 cases (75.9%) and from dehiscent wounds before operation in seven patients (24.1%). Broad-spectrum antibiotics were given, both perioperatively (with specific antibiotics to cover any cultured organism) and post-operatively. In cases where the responsible organism was not known preoperatively, cefazolin or vancomycin was started immediately before surgery and continued for 1 week. This was modified to cover any organism grown preoperatively or from cultures taken at operation, on the basis of an antibiogram. Bacteriology was available in 23 of 29 cases. Among Gram-negative organisms isolated (eight cases, 27.6%), *Pseudomonas aeruginosa* was cultured in six cases and in two cases in association with *Escherichia coli*. Two patients with *Pseudomonas aeruginosa* infection experienced amputation: it was impossible to revascularize one patient and amputation followed the bypass excision. One patient underwent bypass excision and replacement with a femoro-tibial bypass in the cephalic vein. Amputation followed the venous graft failure. One patient, after excision of a femoro-popliteal bypass, underwent a new bypass using Omniflow II (Le Maitre company) and muscle flap transposition, with limb salvage. Three patients with a distal bypass infection and dehiscence of a surgical wound were submitted to multiple surgical debridements and various sessions of VAC therapy, with subsequent limb salvage. About the two patients with infection caused by *P. aeruginosa* and *E. coli*, the patient with an axillofemoral bypass received the bypass excision and replacement with a new bypass in a clean field, with limb salvage. The other one, with dehiscence of the groin wound, underwent surgical debridement of the wound and various sessions of VAC therapy, experiencing limb salvage. The majority of germs cultured were Gram-positive (12 cases, 41.4%): Methicillin-resistent *Staphylococcus aureus* (MRSA) in four cases; Methicillin-sensitive *Staphylococcus aureus* (MSSA) in four cases; *Staphylococcus warneri*, *Staphylococcus lugdunensis*, and *Staphylococcus epidermidis*, respectively, in one case; *Enterococcus faecalis* in one case. One patient with infection caused by MRSA experienced amputation, after bypass excision and death during follow-up for different causes. One patient underwent axillo-femoral bypass excision but he did not require revascularization. The patient with an ilio-femoral bypass infection received surgical debridement of the groin wound, VAC therapy application, and bypass coverage with a muscle flap transposition. This patient experienced limb salvage. In the case of femoro-popliteal bypass infection and dehiscence of the groin wound, after surgical debridement of the wound and VAC therapy, the patient healed. About patients with MSSA infection, we recorded an amputation after bypass removal and three-limb salvage: one patient with prosthetic leg infection of an axillo-femoral bypass experienced healing after surgical debridement of the groin wound and VAC therapy; one patient with a femoro-femoral crossover bypass received an axillo-bifemoral bypass after bypass excision; the last patient with a false aneurysm at the distal anastomosis of a femoro-popliteal bypass underwent bypass excision and replacement with a femoro-peroneal bypass in GSV followed by limb salvage. Three patients with a femoro-popliteal bypass infection caused by Staphylococcus lugdunensis, Staphylococcus warneri, and Enterococcus faecalis, respectively, underwent bypass removal and replacement with a femoro-distal bypass in GSV, with limb salvage. The patient with infection of an axillo-femoral bypass caused by *S. epidermidis* experienced limb salvage after graft removal and replacement with a new bypass in a clean field. In three cases, an infection caused by the association of Gram-positive and Gram-negative organisms was recorded. In two cases, we observed an infection caused by MRSA and *P. aeruginosa*: it was impossible to revascularize one patient with a femoro-popliteal bypass infection and she underwent amputation and death for sepsis during her hospital stay; the other one received a venous femoro-distal bypass after prosthesis excision, with limb salvage. A patient with infection of a femoro-popliteal bypass by *Proteus mirabilis* and *Staphylococcus aureus* received graft removal and replacement with Omniflow II associated with a muscle flap transposition. After occlusion of the Omniflow II graft, she underwent amputation and death during follow-up for comorbidities.

In six cases, the germ responsible for the graft infection was not identified. Three of these patients presented a femoro-tibial infected bypass: we recorded two amputations after bypass removal in patients impossible to revascularize. One of these patients underwent surgical debridement of the wound, proximal bypass removal and replacement with Omniflow II, and coverage through a muscle flap transposition, with subsequent limb salvage. One patient with ilio-femoral bypass and development of an abscess on the surgical site received multiple surgical debridements, and VAC therapy followed by bypass coverage through a muscle transposition, with subsequent limb salvage. A patient with a late infection of a femoro-femoral crossover bypass underwent bypass excision without the need for revascularization and subsequent limb salvage.

The last patient presented a proximal false aneurysm of a femoro-femoral crossover bypass: he underwent surgical repair of the false aneurysm with bypass preservation and consequent limb salvage.

**Figure 1:**
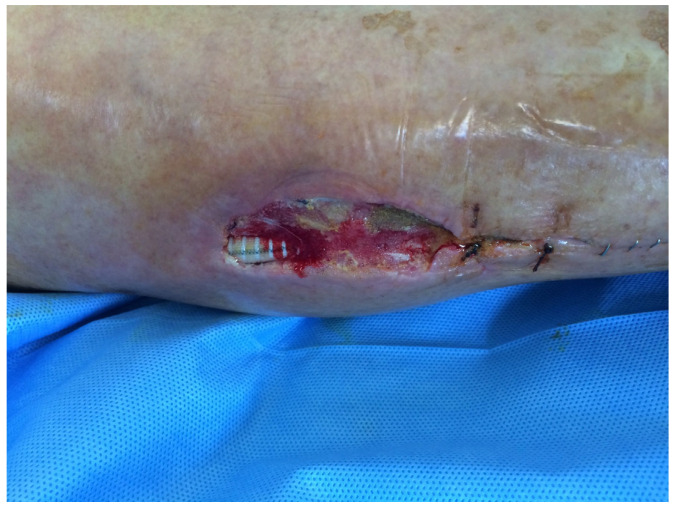
This is a figure that shows surgical wound dehiscence with prosthesis exposure on the right leg, after femoro-peroneal bypass in PTFE.

**Figure 2:**
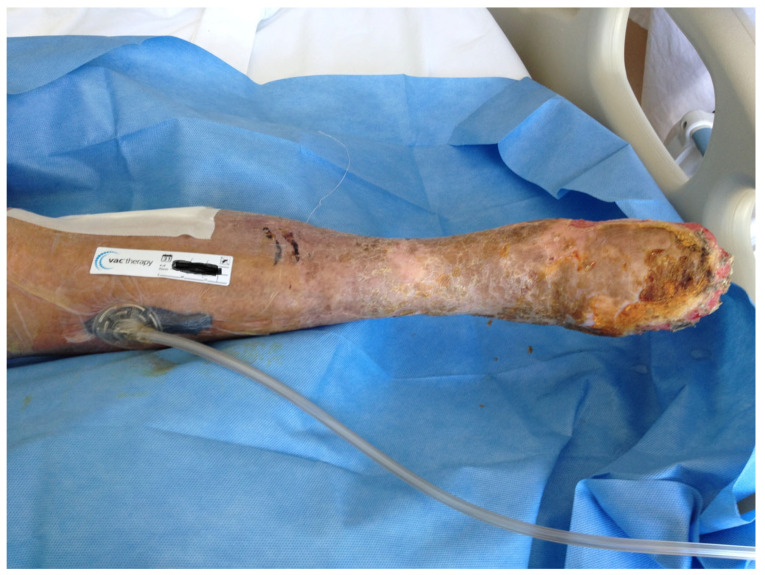
VAC therapy application on the dehiscent wound after surgical wound debridements.

**Figure 3:**
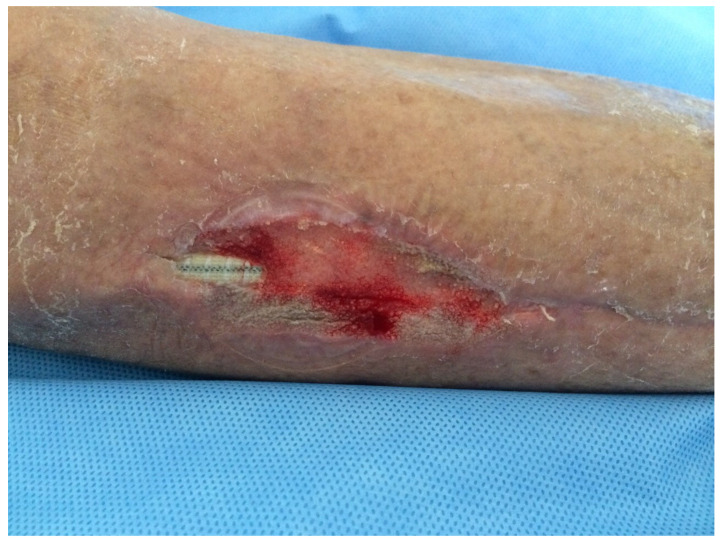
Outcome after 3 sessions of VAC therapy application with almost complete coverage and wound healing.

In conclusion, the primary mode of treatment was represented by prosthesis excision (20 cases, 68.9%: 17 entire prosthesis removals and three partial graft excisions): nine patients received a new bypass; it was impossible to revascularize six patients, and in two cases, the revascularization was not required. Three patients with false aneurysm on the proximal anastomosis underwent removal of the proximal segment of the bypass, replaced using Omniflow II. Among the remaining nine cases (31.1%), one patient with femoro-femoral crossover bypass underwent surgical repair of a proximal false aneurysm with bypass preservation and limb salvage. Eight patients experienced a successful treatment through multiple surgical wound debridements and VAC therapy application (two femoro-popliteal, one ilio-peroneal, one iliofemoral, one femoro-tibial, one femoro-peroneal, and one axillo-femoral bypass infections). (Figure 1, Figure 2 and Figure 3) VAC therapy was usually started the day after the wound surgical revision: a polyurethane sponge (KCI Medical, San Antonio, TX, USA) was applied with a continuous topical negative pressure of 125 mmHg. Changes in dressings were performed every three days, with a medium of five sessions for patients (mean duration of treatment: 15 days). Six patients (20.7%) underwent amputation directly after infected bypass removal; two patients (6.9%) experienced amputation after occlusion of a new bypass replacing the removed infected prosthesis. Five deaths (17.2%) occurred during follow-up, three in patients who underwent amputation: one death occurred during hospital stay for sepsis (blood culture positive for MRSA), while the remaining cases were recorded during follow-up for comorbidities.

## 4. Discussion

PVGI after surgical revascularization for CLI represents a serious complication with high rates of mortality and amputation [1,2,3]. Most graft infections occur in the groin, usually in the early post-operative period, typically as a result of a progressive surgical site infection [1,22]. Comorbid risk factors associated with PVGI include the presence of ischaemic leg ulcers, diabetes, smoking, infected lymph nodes, and transient bacteraemia from a purulent draining wound or sinus, an abscess, a lymphocele, skin necrosis, pain, septic emboli with petechia, a pulsatile mass, fever, or graft thrombosis [1,14,23,24,25,26]. Actually, there is no consensus on the diagnostic criteria or on best management of PVGI. Antibiotic therapy is the first stage of management of PVGI, followed by prosthesis surgical removal and reconstruction with autologous material. In the absence of appropriate venous graft material, prosthetic grafts with a potentially higher resistance to reinfection (e.g., silver bonded or rifampicin bonded) can be used alternatively, although reinfection rates are higher compared to venous grafts [27] and long-term patency is significantly lower than in venous bypasses. However, experiences with the use of biosynthetic grafts as graft material in elective infrainguinal revascularization procedures have shown acceptable secondary patency and limb salvage rates [28]. In particular, Omniflow I presented higher early occlusion rates due to the initial thrombogenicity and the disposition to aneurysmal degeneration that impeded its widespread clinical use [29]. The improved collagen structure of the second-generation graft (Omniflow II, Bio Nova International, Victoria, Australia) is supposed to increase wall strength and stability to reduce aneurysmal changes. Remarkably, the infection rate of biosynthetic grafts in elective femoropopliteal reconstructions is very low [30].

Topel et al. [31] reported seven cases of infected infrainguinal prosthetic grafts that were explanted and replaced by Omniflow II^®^, in the absence of a suitable peripheral vein. They did not record early or late reinfection or amputations, with graft occlusion in three cases and one death from pneumonia 11 months post-operatively.

Chaudhry [32] published a case of a mycotic aneurysm of the common femoral artery successfully reconstructed with an Omniflow II prosthesis in 2008. Another group reported a patient with an infected prosthetic infrainguinal graft, which was also replaced with an Omniflow II prosthesis [33]. In both cases, no reinfection occurred.

A less invasive alternative to prosthesis removal is represented by the conservative treatment, with surgical debridement of the wound and VAC therapy application, with or without muscle transposition of coverage. As already described in other districts [34,35], VAC therapy has been reported to have several beneficial effects on wound healing, such as creating a moist wound-healing environment, drainage of superfluous fluid, reduction in tissue oedema, cleansing deep wounds from bacteria, accelerating the formation of vascularized granulation tissue, and faster approximation of wound edges [36,37].

However, experience with VAC therapy for infected bypasses in the lower limb is limited [38].

Acosta et al. [39] described 28 synthetic graft infections treated by VAC therapy for a mean period of 20 days. Two serious bleeding episodes from the suture lines occurred. The proportion of patent bypass grafts was 91% at a median time of 3.5 months from the start of the treatment. Five patients with seven bypasses had persistent infection or re-infection, and the total graft preservation rate was 76%.

Mayer et al. [19] reported the outcomes of 44 polymorbid patients with Szilagyi III infections. Forty grafts (prosthetic = 24, vein = 3, biological = 13) and nine native arteries were involved. VAC therapy was applied directly on grafts/arteries after radical debridement of infected tissue with a median duration of 33 days (IQR: 20–78), and a hospital stay of 32 (IQR: 20–82) days. One-year mortality was 16% (7/44). Long-term mortality after a mean follow-up of 43 months (SD: 21) was 41% (18/44). Complete wound healing was achieved in 91% (40/44). In 37 of 44 patients, grafts were preserved long-term without reinfection.

In our institution, among 196 prosthetic bypasses performed between 2016 and 2021, 16 (8.2%) presented a PVGI. In the same period, thirteen other patients who submitted to prosthetic peripheral bypass in other centers presented at our unit with a PVGI. So, a total of 29 patients underwent multimodal treatments for PVGI in our institution. The primary mode of treatment was represented by prosthesis excision (20 cases, 68.9%: 17 entire prosthesis removals and three partial graft excisions): nine patients received a new bypass; it was impossible to revascularize six patients with redo-bypass after multiple attempts of lower limb revascularization, and they underwent amputation directly; in two cases, the revascularization was not required. Three patients with false aneurysm on the proximal anastomosis underwent removal of the proximal segment of the bypass, replaced using Omniflow II, accompanied by muscle transposition of coverage. More recently, we returned to VAC therapy after surgical debridements of the dehiscent wound in eight patients (27.6%). During follow-up, we recorded five deaths (17.2%), of which only one related to graft infection, and eight (27.6%) amputations. The amputations occurred in patients with a history of multiple attempts of revascularization: six patients (20.7%) underwent amputation directly after bypass excision, while two patients (6.9%) underwent amputation after a last attempt of revascularization. In accordance with the literature data [40] the analysis of the data collected in our institution suggests that PVGI in patients with redo-bypasses after failure of multiple attempts of revascularization and the presence of advanced gangrene and active infection at the time of operation (Rutherford 6 class) represent an important risk factor for amputation [41]. In addition, the serious comorbidities recorded in patients affected by CLI are often responsible for the death.

Cherry et al. [42] reviewed 39 infected lower extremity bypasses (33 prosthetic bypass, four venous bypasses, and composite in two). Twenty-eight infected grafts were treated with complete graft removal and nine new grafts were placed. Recurrent infection developed in five cases, and two patients died of complications of graft infection. Eleven patients with patent bypasses were treated without graft excision: muscle transposition in five cases, incision and drainage of abscesses in five cases, and excision of a persistent sinus tract in one case. One patient underwent major amputation 6.3 years after treatment of graft infection. Limb salvage was significantly higher (*p* = 0.012, log-rank test) than in patients treated with graft excision. One patient died, and no recurrent infections developed.

Siracuse et al. [40] reported their experience of 496 prosthetic grafts with a graft infection rate of 3.8% (19) at a mean follow-up of 27 months. Multivariable analysis showed that redo bypass, active infection at the time of bypass, female gender, and diabetes mellitus were significant predictors of graft infection. Graft infection was predictive of major lower extremity amputation (HR, 9.8; 95% CI, 3.5–27.1), as was preoperative tissue loss (HR, 4.7; 95% CI, 1.8–11.9). Infected grafts were removed 79% of the time. In the first 7 years of this study, all grafts except one were removed. However, in the latter 3 years, 50% were preserved by debridement with placement of a VAC device, with or without local rotational muscle flap closure.

Zetrenne et al. [2] described 45 cases of PVGI with 11 amputations (24%) and three perioperative deaths related to graft infection; the other eight deaths were from various unrelated causes. Overall, there was a 6% perioperative mortality rate in this series. The primary mode of therapy was muscle flap transposition (18 cases, 40%), alone (n = 15) or with in situ graft replacement (n = 3). Ten (67%) of 15 grafts were preserved with muscle flap transposition. Samson group 3 (n = 30) cases were treated mostly by graft excision (n = 16; 53%), and fewer than half were preserved (n = 14; 46%).The Samson group 4 (n = 9) and group 5 (n = 6) cases were managed with a variety of surgical treatments: ex situ bypass (n = 4; 27%), graft excision alone (n = 3; 20%), muscle flaps (n = 3; 20%), and in situ bypass with muscle flap (n = 2; 13%). Additionally, irrigation and debridement according to Kwaan and Connolly’s method [12] were performed on three patients (20%).

## 5. Conclusions

In conclusion, PVGI represents a very serious complication after lower extremity bypass for CLI. Redo-bypass, active infection at the time of bypass, and advanced gangrene are associated with a higher risk for PVGI and major extremity amputation. Complete graft removal and replacement by venous material or Omniflow II represents the typical treatment. However, aggressive local treatment including drainage, debridement, VAC therapy application, and muscle transposition seems to be a better solution in selected patients without the need for graft removal and with rates of limb salvage superior to those obtained with excisional therapy. In our experience, nonexcisional therapy of infected peripheral grafts is a good and sometimes better strategy for limb salvage rate, reinfection, and mortality rates than traditional excisional treatment, with or without reconstruction.

A global evaluation in patients with critical lower limb ischemia, very tissue loss, and local infection should take into account an aggressive prophylatic local and systemic antibiotical therapy to prevent a systemic infection. Prospective, multicenter, well-designed randomized clinical trials are needed to better understand for which patient a conservative approach should be recommended

## Figures and Tables

**Table 1:** Demographic characteristics, clinical status, germs involved, treatment, and outcomes of 29 patients with peripheral prosthetic vascular graft infection.

Patient Sex, Age	Type of Bypass	Type of Graft	Time, Type and Location of Infection	Organism. Site of Bacteriological Sampling	Surgical Treatment	Antibiotic Treatment	Outcome
1, M 82 y	Right axillo-femoral bypass	Dacron	Late infection. Abscess along the abdominal wall	*Staphylococcus epidermidis.* Prosthesis	Bypass excision. New bypass in a clean field	Tigecycline, gentamicin	Limb salvage
2, F 72 y	Right ilio-tibial bypass (redo-bypass)	PTFE	Early infection. Abscess and surgical wound dehiscence with pus discharge	MSSA. Prosthesis, dehiscent groin wound	Bypass excision. Impossible to revascularize	Ceftriaxone, amikacin	Amputation
3, M 78 y	Left ilio-peroneal bypass (redo-bypass)	PTFE	Early infection. Leg abscess with surgical wound dehiscence	*Pseudomonas* *aeruginosa.* Prosthesis, dehiscent leg wound. Negative blood culture	Surgical debridement of the dehiscent wound. VAC therapy. Surgical closure	Piperacillin-tazobactam	Limb salvage
4, F 81 y	Right femoro-tibial bypass (redo-bypass)	PTFE	Early infection. Graft occlusion	*Pseudomonas aeruginosa.* Prosthesis, dehiscent groin wound	Bypass excision. Impossible to revascularize	Amikacin	Amputation
5, M 61 y	Left femoro-tibial bypass (redo-bypass)	PTFE	Early infection. Proximal false aneurysm	Sterile. Prosthesis	Proximal bypass excision and replacement with Omniflow II	Ceftazidime, gentamicin	Limb salvage
6, F 79 y	Left femoro-tibial bypass (redo-bypass)	PTFE	Early infection. Bleeding	Sterile. Prosthesis.	Bypass excision. Impossible to revascularize	Ceftazidime	Amputation
7, M 77 y	Right femoro-popliteal bypass	PTFE	Late infection. Groin suture dehiscence with persistent pus discharge	MRSA (dehiscent groin wound); Pseudomonas aeruginosa (prosthesis)	Bypass excision and replacement in GSV	Imipenem, ciprofloxacin	Limb salvage
8, M 76 y	Left ilio-femoral bypass	PTFE	Early infection. Abscess	Sterile (periprosthetic material)	Surgical site debridement and pus evacuation. VAC therapy. Muscle flap transposition	Ceftriaxone	Limb salvage
9,M 78 y	Right femoro-popliteal bypass	PTFE	Late infection. Acute ischemia	*Staphylococcus lugdunensis.* Prosthesis	Bypass excision and femoro-peroneal bypass in GSV	Amoxicillin clavulanic acid, Gentamicin	Limb Salvage
10, M 78 y	Femoro-tibial bypass (redo-bypass)	PTFE	Early infection. Bleeding	MRSA. Prosthesis and dehiscent groin wound	Bypass excision. Impossible to revascularize	Teicoplanin, vancomycin	Amputation. Death during follow-up
11, M 75	Femoro-femoral crossover bypass	PTFE	Late infection. Graft occlusion	Sterile (prosthesis)	Bypass excision. Revascularization not required	Vancomycin, rifampicin	Limb salvage
12, M 69 y	Right femoro-tibial bypass (redo- bypass)	PTFE	Early infection. Surgical wound dehiscence with persistent discharge	Sterile (prosthesis)	Bypass excision. Impossible to revascularize	Ceftazidime	Amputation
13, F 78 y	Left femoro-popliteal bypass	PTFE	Early infection. Bleeding	*Enterococcus faecalis.* Prosthesis and dehiscent groin wound	Bypass excision. Femoro-peroneal bypass in GSV	Piperacillin-tazobactam, amikacin	Limb salvage
14, M 67 y	Right femoro-popliteal bypass	PTFE	Late infection. Proximal false aneurysm	*Pseudomonas aeruginosa.* Prosthesis, dehiscent groin wound	Proximal bypass excision and replacement in Omniflow II with muscle flap transposition.	Ampicillin sulbactam	Limb salvage
15, M 67 y	Left axillo-femoral bypass (Dacron)	Dacron	Late infection. Seroma	MRSA. Prosthesis	Bypass excision and femoral artery patch in GSV. Revascularization not required	Teicoplanin, vancomycin	Limb salvage
16, M 82 y	Right axillo-femoral bypass (Dacron)	Dacron	Late infection. Seroma	*Escherichia coli* (periprosthetic fluid), *Pseudomonas aeruginosa* (prosthesis)	Bypass excision and replacement in a clean field	Amikacin, colistimethate sodium	Limb salvage. Death during follow-up
17, F 68 y	Right femoro-popliteal bypass	PTFE	Early infection. Acute ischemia	MRSA (blood culture). *Pseudomonas aeruginosa* and *Staphylococcus aureus* (prosthesis)	Bypass excision. Impossible to revascularize	Meropenem, colistimethate sodium	Amputation. Death during hospital stay for sepsis
18, F 65 y	Left femoro-popliteal bypass	PTFE	Late infection. Proximal false aneurysm	*Proteus mirabilis* (dehiscent groin wound). MSSA (dehiscent thigh wound)	Proximal bypass excision and replacement in Omniflow II. Muscle flap transposition. Graft occlusion	Ertapenem	Amputation after new bypass occlusion. Death during follow-up
19,F 71 y	Right femoro-popliteal bypass	PTFE	Early infection. Acute ischemia and peripheral septic arterial embolism on the skin	*Staphylococcus warneri* (thrombus and prosthesis)	Bypass excision and femoro-peroneal bypass in GSV	Teicoplanin	Limb salvage
20, M 65 y	Femoro-femoral crossover bypass	PTFE	Early infection. Proximal false aneurysm	MSSA. Prosthesis	Bypass excision and axillo-bifemoral bypass	Rifampicin, trimethoprim/sulfamethoxazole, daptomycin	Limb salvage
21, F 48 y	Left Ilio-tibial bypass (redo-bypass)	PTFE	Early infection. Surgical wound dehiscence with persistent discharge	*Pseudomonas aeruginosa.* Prosthesis	Bypass excision and replacement in cephalic vein. Graft occlusion	Amikacin	Amputation after new bypass occlusion
22, F 83 y	Right axillo-femoral bypass in Dacron	Dacron	Late infection. Groin surgical wound dehiscence with persistent discharge	MSSA. Dehiscent groin wound	Surgical debridement of the dehiscent groin wound. VAC therapy. Surgical closure of the wound	Meropenem	Limb salvage
23, M 80 y	Right femoro-popliteal bypass	PTFE	Early infection. Groin surgical wound dehiscence with persistent discharge	*Escherichia coli, Pseudomonas aeruginosa.* Dehiscent groin wound	Surgical debridement of the dehiscent groin wound. VAC therapy. Surgical closure of the dehiscent wound	Amikacin, colistimethate sodium	Limb salvage
24, F 77 y	Left ilio-femoral bypass	PTFE	Early infection. Surgical wound dehiscence with persistent discharge	MRSA	Surgical debridement, VAC therapy. Muscle flap transposition.	Teicoplanin, vancomycin	Limb salvage
25, M 83 y	Left femoro-popliteal bypass	PTFE	Early infection. Surgical wound dehiscence with persistent discharge	MRSA	Surgical debridement of the dehiscent groin wound and VAC therapy. Surgical closure of the dehiscent wound	Teicoplanin, vancomycin	Limb salvage. Death during follow-up
26, F 79 y (Figure 1, Figure 2 and Figure 3)	Right femoro- peroneal bypass (redo-bypass)	PTFE	Early infection. Dehiscence of the distal surgical wound with prosthesis exposure	*Pseudomonas aeruginosa.* Periprosthetic material	Surgical debridements and VAC therapy	Piperacillin-tazobactam	Limb salvage
27, M 73 y	Femoro-femoral crossover bypass	PTFE	Late infection. Proximal false aneurysm	Sterile Periprosthetic material	Surgical repair	Ceftriaxone	Limb salvage
28, M 76 y	Left femoro-tibial bypass (redo-bypass)	PTFE	Early infection. Dehiscence of the proximal surgical wound with prosthesis exposure	*Pseudomonas aeruginosa.* Periprosthetic fluid	Surgical debridements and VAC therapy	Piperacillin-tazobactam	Limb salvage
29, M 74 y	Right femoro-popliteal bypass	PTFE	Late infection. Proxiaml false aneurysm	MSSA. Prosthesis	Bypass excision and replacement with a femoro-peroneal bypass in GSV	Rifampicin, trimethoprim/sulfamethoxazole, daptomycin	Limb salvage

M = male, F = female, MSSA = Methicillin-sensitive *Staphylococcus aureus*; MRSA = Methicillin-resistant *Staphylococcus aureus*; PTFE = polytetrafluoroethylene.

**Table 2:** Type of infected vascular bypass treated in our institution.

Vascular Bypass and Type of Prosthesis Implanted	In our Institution (N, %)	In Other Institutions
Axillo-femoral (Dacron)	2 (4.9)	2
Femoro-femoral crossover (PTFE)	1 (2.3)	2
Ilio-femoral (PTFE)	1 (3.7)	1
Femoro-popliteal (PTFE)	2 (4.9)	8
Redo-bypass after failure of multiple attempts of revascularization (PTFE): -Ilio-tibial -Femoro-tibial	10 (22.7) 3 7	
TOT = 29	16 (8.2)/196	13

**Table 3:** Demographic localization, surgical treatment, limb outcome, and mortality of 29 patients.

Number	Type of Bypass	Surgical Treatment	Limb Outcome	Mortality
4	Axillo-femoral bypass	2 Bypass excisions and new reconstruction 1 Bypass excision. Revascularization not required 1 Surgical debridement of the dehiscent groin wound.VAC therapy. Surgical closure of the wound	Limb salvage Limb salvage Limb salvage	
2	Ilio-tibial bypass	1 Bypass excision. Impossible to revascularize 1 Bypass excision and replacement in cephalic vein.	Amputation Amputation after new bypass occlusion	
1	Ilio-peroneal bypass	Surgical debridement of the dehiscent wound. VAC therapy. Surgical closure	Limb salvage	
6	Femoro-tibial bypass	4 Bypass excisions. Impossible to revascularize 1 Proximal bypass excision and replacemnt with Omniflow II 1 Debridements and VAC therapy	Amputation 1 Limb salvage—1 Amputation Limb salvage	1 1
10	Femoro-popliteal bypass	5 Bypass excisions and replacement in GSV 1 Bypass excision. Impossible to revascularize 2 Proximal bypass excisions and replacement in Omniflow II with muscle flap transposition. 2 Surgical debridements of the dehiscent groin wound. VAC therapy.	Limb salvage Amputation Limb Salvage Amputation Limb salvage	1 1 1
2	Ilio-femoral bypass	Surgical site debridement. VAC therapy. Muscle flap transposition	Limb salvage	
3	Femoro-femoral crossover bypass	Bypass excision. Revascularization not required Bypass excision and axillo-bifemoral bypass Surgical repair	Limb salvage Limb salvage Limb salvage	
1	Femoro-peroneal bypass	Surgical debridements and VAC therapy	Limb salvage	

## Data Availability

Data are unavailable due to privacy and institutional restrictions.
